# Change of Th17 Lymphocytes and Treg/Th17 in Typical and Atypical Optic Neuritis

**DOI:** 10.1371/journal.pone.0146270

**Published:** 2016-01-19

**Authors:** Hengri Cong, Hanqiu Jiang, Jingting Peng, Shilei Cui, Lijuan Liu, Jiawei Wang, Xiaojun Zhang

**Affiliations:** 1 Department of Neurology, Beijing Tongren Hospital, Capital Medical University, Beijing, 100730, China; 2 Eye Center, Beijing Tongren Hospital, Capital Medical University, Beijing, 100730, China; Wayne State University, UNITED STATES

## Abstract

**Background:**

Typical and atypical optic neuritis (ON) are two clinical types of autoimmune inflammatory diseases of the optic nerve that causes acute vision loss, and are difficult to distinguish in their early stages. The disturbance in the balance of Th17 and Treg lymphocytes is thought to play an essential role in these autoimmune inflammatory diseases.

**Objectives:**

To detect the clinical relevance of Th17 and Treg in peripheral blood and the ratio of Treg/Th17 in patients with typical and atypical ON. To determine whether analysis of Th17 and Treg lymphocytes will provides insights into the different disease phenotypes of typical and atypical ON.

**Methods:**

We studied a consecutive series of patients aged 14–70 years who presented to our neurological department with typical ON (n = 30) or atypical ON (n = 33) within 4 weeks of their acute attacks. Routine clinical tests and ophthalmological examination were performed in all patients. Blood samples were collected from untreated patients and from gender- and age-matched healthy controls (n = 30). The proportion of peripheral blood Th17 cells and Treg cells was determined by flow cytometry.

**Results:**

Patients with atypical ON had a higher proportion of Th17 cells than patients with typical ON (3.61±1.56 vs 2.55±1.74, *P<*0.01) or controls (1.45±0.86, *P<*0.01). The proportion of Th17 cells in patients with typical ON was also markedly higher than in controls (*P<*0.01). The mean percentage of Treg cells in atypical ON (6.31±2.11) and typical ON (6.80±2.00) were significantly lower when compared to controls (8.29±2.32, both *P<*0.01). No significant difference in Treg frequency was observed between typical ON and atypical ON (*p*>0.05).

**Conclusions:**

The frequency of Th17 cells is higher in atypical ON than typical ON, and patients with atypical ON have a greater imbalance of pro-inflammatory and regulatory cells than patients with typical ON when compared with controls. These changes are indicative of distinct pathological mechanisms and may provide useful information to distinguish typical and atypical ON.

## Introduction

Optic neuritis (ON) refers to conditions that involve inflammation of the optic nerve [1]. It has been previously demonstrated that acute inflammatory demyelinating ON (IDON) has a close relationship with multiple sclerosis (MS). Approximately 20% of MS patients will initially present with IDON, with 50% of MS patients developing IDON during the course of their disease [2]. As IDON is commonly observed in the early stages of MS, especially in Western countries, and this form of ON is usually referred to as “typical ON”. ON with different etiologies other than MS are termed as “atypical ON”. Atypical ON commonly presents as an early manifestation of neuromyelistis optica (NMO) or NMO spectrum disorder (NMOSD) [3, 4]. These latter forms of ON are common in Asia [4–6], and differ from typical ON with respect to mechanism, treatment strategies, and ultimate neurological outcomes [4, 5]. It is, therefore, crucial to distinguish between typical and atypical ON, especially in the early stages of presentation. Due to their clinically overlapping characteristics, a reliable biomarker is needed to distinguish between typical and atypical ON. AQP4 antibody (AQP4-Ab) has been suggested, but prior studies indicated that only 9–34% of ON related to NMO patients were AQP4-Ab positive [1, 7, 8]. This low sensitivity of AQP4-Ab underscores the importance of identifying an alternative.

In the present study, we examine the relative balance of T helper (Th) cell subpopulations to distinguish between typical and atypical ON. CD4+ Th cells are an essential component of the immune system. While CD4+ Th cells have historically been divided into Th1 and Th2 subsets, several additional Th cell subpopulations have been described. Of these, IL-17 producing (Th17) cells and FoxP3+ regulatory (Treg) cells are most prominent, and both are believed to play an essential role in human autoimmune disease[6, 9]. Recent studies have showed that the imbalanced Th17 is related to both MS and NMO [10–14], but to our knowledge, this relationship has not yet been investigated in ON patients. In view of the opposing immune functions of Th17 and Treg cells, the plasticity of Th17/Treg differentiation, their important roles in autoimmune disease, and the distinct clinical phenotypes of typical and atypical ON, we compared the relative expression of these cells in these two forms of ON. To accomplish this goal, we determined the number of Th17 cells (CD3+CD4+IL-17A+ Th cells) and Treg cells (CD4+CD25+FoxP3+ Th cells) in the peripheral blood, and the ratio of these two cell types.

## Methods

### Ethics statement

This research project was approved by the Medical Ethics Committee of the Beijing Tongren Hospital, Capital Medical University in accordance with the principles stated in the Declaration of Helsinki. All participants provided their written informed consent before the start of the research. For child participants, we obtained consent from the patients and their guardians. Written consent was obtained from the next of kin, caretakers, or guardians on behalf of the children enrolled in our study. And the ethics committee approves this consent procedure.

### Patients and controls

We examined a series of consecutive patients presenting to the Department of Neurology, Beijing Tongren Hospital, Capital Medical University between November 2014 and April 2015. We recruited patients between the ages of 14 and 70 years who were seen within one month of ON onset, either as a first attack or an attack with a recurring ON phenotype. None of the patients had received immunomodulatory or immunosuppressive treatment within 6 months prior to the study. A total of 30 typical and 33 atypical ON patients were enrolled, based on the diagnosis criteria of ON [1]. Subjects with other central nervous system conditions or with systemic infections were excluded. Best-corrected visual acuity (VA) was measured and reported, where values of 1.0 and 0.1 correspond to acuity of 20/20 and 20/200, respectively.

A total of 30 gender- and age-match healthy volunteers were recruited as control subjects. No control subject had a history of autoimmune disorders.

### Preparation and processing of blood samples

Blood samples were collected in sodium heparinized tubes from each ON patient at their initial presentation, and also from control subjects. To blind our analyses, blood samples were assigned a unique code number.

To detect Th17 cells, fresh heparinized peripheral blood (500 μl) was stimulated with PMA (100 ng/ml) and ionomycin (1μg/ml, Sigma), in the presence of Brefeldin A (100 μg/ml, Becton Dickinson) for 5 h. After this stimulation period, cells were harvested and washed with PBS. Erythrocytes were then lysed with (FACS) lysing solution (BD PharMingen) and leukocytes were washed once in Flow Cytometry Staining Buffer (eBioscience, USA). Then cells were stained with anti-CD4-APC Abs (BD Biosciences, USA) and anti-CD3-PerCP Abs (BD Biosciences, USA). After surface staining, cells were blocked, fixed, and permeabilized using Fix & Perm (eBioscience, USA) according to the manufacturer’s instructions. Cells were then were incubated with PE-labeled anti–IL-17 Abs (BD Biosciences, USA) and relevant isotype controls (BD Biosciences, USA), and then analyzed by flow cytometry [15] (FACSCalibur; BD Bioscience, USA).

To identify Treg cells, fresh heparinized peripheral blood was labeled with anti-CD4-FITC Abs and anti-CD25-APC Abs and their respective isotype controls. Surface labeling was followed by fixation and permeabilization using Fix & Perm. The cells were then incubated with anti-FOXP3-PE antibody and their isotype controls, and then analyzed by flow cytometry [16]. All antibodies were purchased from BD Biosciences (USA) except for anti-FOXP3-APE antibody (eBioscience, USA).

### Statistical interpretation

Statistical Package for Social Sciences (SPSS for Windows, version 17.0) was used for statistical analysis. The statistical differences between control and ON patient groups were evaluated using one-way ANOVA and Nonparametric Tests. Data are described as mean ± standard error of the mean. Pearson’s and Spearman’s rank correlations were used to assess the relationships between variables. A *P* value < 0.05 represented a significant result for all statistical tests.

## Results

### Demographic and clinical features of patients and controls

Table 1 describes the demographics of the 30 patients enrolled with typical ON (20 women, 10 men), the 33 patients enrolled with atypical ON (23 women, 10 men) and the 30 healthy subjects who comprised control C group (21 women, 9 men). There was no significant difference in the gender (*P*>0.05) or age (*P*>0.05) of the subjects in these three groups. Most patients with typical ON had no prior ON attack 26/30, while 16/33 patients with atypical ON had a history of ON and this difference was statistically significant (*P*<0.05). VA was severely reduced at the initial visit for both typical and atypical ON (*P*<0.05). While only atypical ON patients were AQP4 positive, the AQP4 positive rate was significantly higher in atypical ON patients than in typical ON patients (16/33 vs. 0/30, *P*<0.05), and we also noted that this analysis only detected nearly half of atypical ON, in consistent with prior studies [1].

**Table 1:** Demographic and clinical features of patient cohorts and controls.

Patients’ information	Typical ON	Atypical ON	Control
Number	30	33	30
Age (years)	34.90±15.02	41.82±16.23	37.27±13.10
Gender (male/female)	10/20	10/23	9/21
First attack / Relapse	26/4	17/16	NA
Days post-attack	18.94±18.55	17.20±8.76	NA
Visual Acuity (VA)	0.29±0.40	0.12±0.25	ND
AQP4-Ab			
Positive	0	16	ND
Negative	30	17	ND

First attack: the first time the patients had ON attack; Relapse: patients had prior history of ON

ON: Optic Neuritis, NA: Not applicable; ND: Not done; AQP4-Ab: presence of autoantibody to aquaporin 4. There was no significant difference in gender (*P*>0.05) or age (*P*>0.05) between these three groups. There was no significant different in VA between typical and atypical ON (*P*>0.05). Patients with atypical ON had a significantly higher rate of relapse (*P<*0.05) and of being AQP4 positive (*P<*0.05).

### Th17 cells in typical ON, atypical ON and control subjects

**Fig 1:**
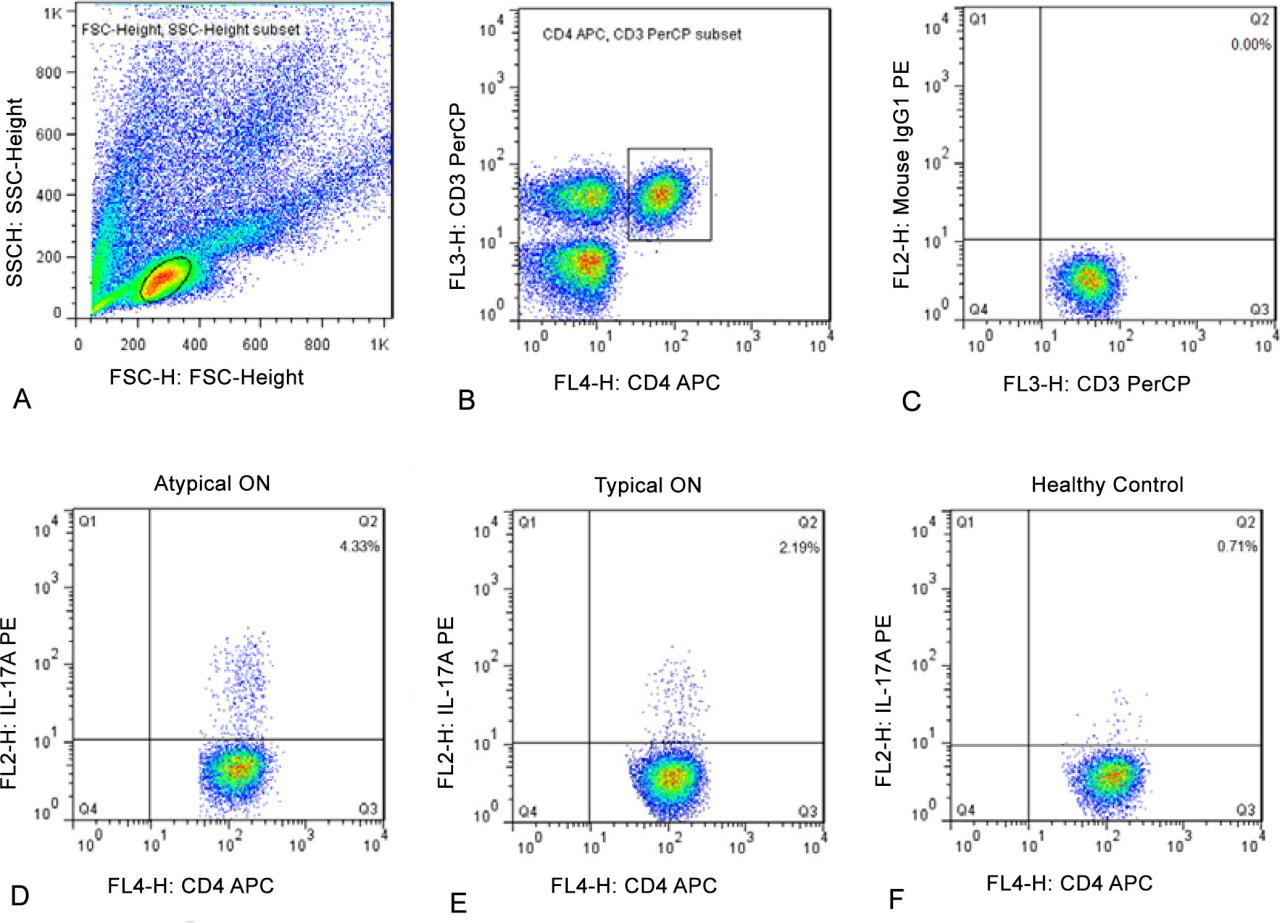
Proportion of CD3+CD4+IL-17A Th17 cells in atypical ON, typical ON and HCs. Lymphocyte (A), CD3+CD4+ T Cells (B), and Isotype control staining of IL-17A (C) in a representative subject. Th17 cells from representative patients with atypical ON (D) or typical ON (E), and from a healthy control subject (F). Note that the proportion of Th17 cells is highest in the atypical ON patient (D), intermediate in the typical ON patient (E) and lowest in the control subject (F).

**Fig 2:**
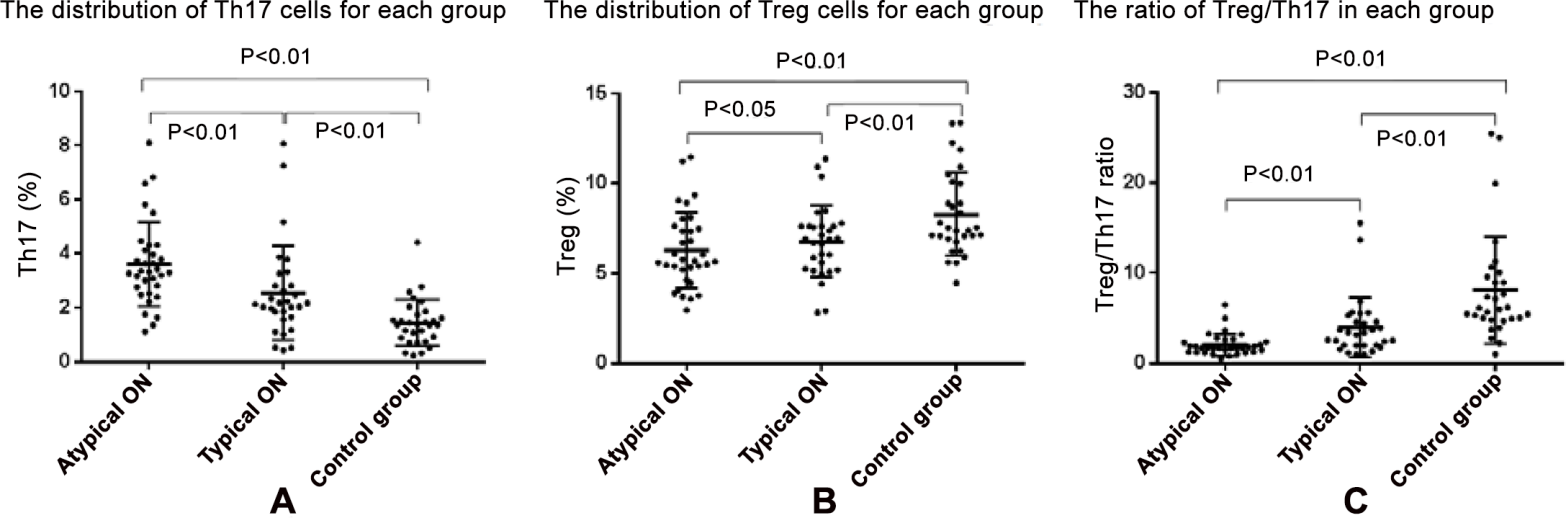
A. The percentage of CD3+CD4+IL-17A+ Th17 cells in patients with atypical ON (left) or typical ON (middle), and from control subjects (right). The percentage of Th17 cells was higher in atypical ON patients than in typical ON patients (*P<*0.01) and controls (*P<*0.01). The percentage of Th17 cells in patients with typical ON was significantly higher than in control subjects (*P<*0.01). B. The percentage of CD4+CD25+FoxP3+ Treg cells in patients with atypical ON (left) or typical ON (middle), and from control subjects (right). The percentage of Treg cells was significantly lower in atypical ON patients or patients with typical ON than in controls (both *P<*0.01). The number of Treg cells did not statistically differ between patients with typical ON compared to atypical ON patients (*P*>0.05).C. The ratio of Th17/Treg lymphocytes in patients with atypical ON (left) or typical ON (middle), and from control subjects (right). Atypical ON patients had significantly lower ratios of Treg/Th17 than patients with typical ON (*P<*0.01). The Treg/Th17 ratio was significantly lower in both patient groups than in control subjects (*P<*0.01 for both comparisons).

Th17 cells were identified based on their expression of CD3 and CD4 surface markers and IL-17A intracellular markers. As shown in Fig 1, lymphocytes for each subject were initially sorted based on their side scatter (SSC) and forward scatter (FSC) fluorescent signals (Fig 1A), and then based on their expression of CD3/CD4 surface markers (Fig 1B) and their expression of isotype control staining of IL-17A (Fig 1C) and IL-17 (Fig 1D–1F). Fig 1D–1F compares plots of Th17 cells obtained in this manner in a representative patient with typical ON (Fig 1E), with atypical ON (Fig 1D), and also in a healthy control subject (Fig 1F). This analysis demonstrated that atypical ON patients had a higher proportion of Th17 cells than typical ON patients or control subjects. In addition, the proportion of Th17 cells was higher in the typical ON patients than that in control subjects. Fig 2A plots the distribution of Th17 cells for all subjects studied. The percentage of cells that were Th17-positive was higher in atypical ON patients (3.61±1.56) than in typical ON patients (2.55±1.74; *P<*0.01). The percentage of Th17 cells in patients with typical ON was significantly higher than in controls (1.45±0.86; *P<*0.01). There was no significant difference in the frequency of Th17 cells in patients presenting with their first attack of ON and those with a history of ON attacks (3.01±1.90 vs. 3.32±1.25; *P*>0.05).

### Treg cells in typical ON, atypical ON and control subjects

**Fig 3:**
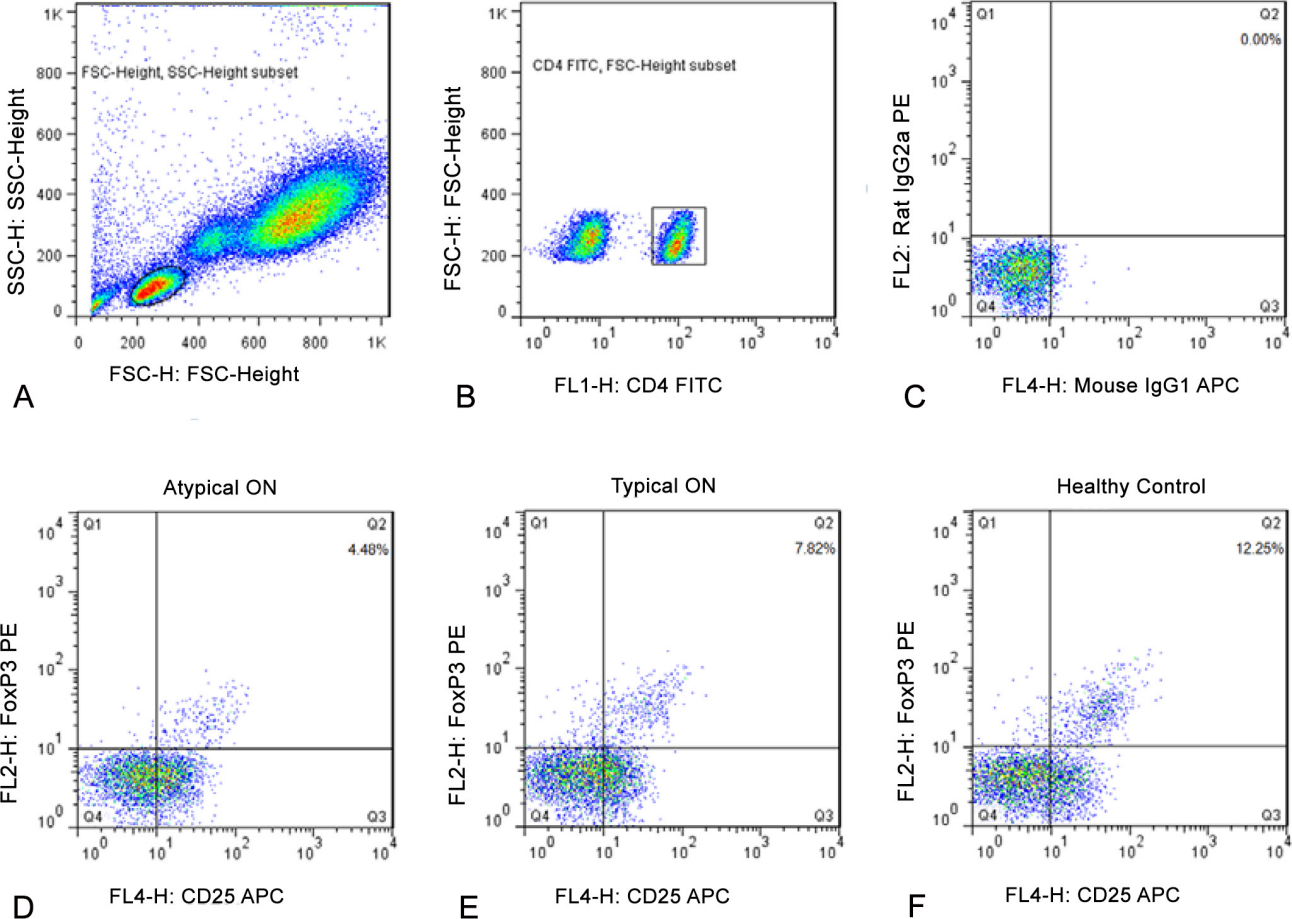
Proportion of CD4+CD25+FoxP3+ Treg cells in atypical ON, typical ON and controls. Lymphocyte (**A**), CD4+ T Cells (**B**), Isotype control staining of CD25 and FoxP3 (**C**), in a representative subject. Treg cells from representative patients with atypical ON (**D**) or typical ON (**E**), and from a healthy control subject (**F**). Note that the proportion of Treg cells is lowest in the atypical ON patient (D), intermediate in the typical ON patient (E) and highest in the control subject (F).

Treg lymphocytes were isolated from each subject were initially sorted based on their SSC and FSC signals (Fig 3A), and then based on their expression of CD4 surface markers (Fig 3B) and their isotype control staining of CD25 and FoxP3 (Fig 3C). Fig 3D–3F compares plots of Treg cells in a representative patient with typical ON (Fig 3E), atypical ON (Fig 3D) and also in a control subject (Fig 3F). It can be noted that, the proportion of Treg cells is lowest in the atypical ON patient, intermediate in the typical ON patient and highest in the control subject. Fig 2B plots the distribution of Treg cells for all subjects studied. Compared with the control group (8.29±2.32), the percentage of Treg cells was significantly lower in both atypical ON patients (6.31±2.11) and patients with typical ON (6.80±2.00) (both *P<*0.01). Although Th17 subset frequency are significantly elevated in atypical ON patients in comparison with typical ON patients, there was no difference in the number of Treg cells between patients with typical ON (6.80±2.00) and atypical ON (6.31±2.11; *P*>0.05).

### Treg/Th17 ratio in typical ON, atypical ON and control subjects

Although significant group differences were noted between typical and atypical ON patients with respect to the density of Th17 and Treg cells, there was substantial overlap between the two groups (Fig 2A and 2B). As the commitment of lineage between Th17 and Treg cells are of opposite directions, with typical ON patients having on average fewer Th17 and more Treg cells than atypical ON patients, we calculated the ratio of Treg and Th17 cell populations in each patient. Fig 2C plots the individual values of the Treg/Th17 ratio for each subject studied. The values for patients with atypical ON are significantly lower than those with typical ON. When the group data are considered, atypical ON patients had significantly (*P<*0.01) lower values of the Treg/Th17 ratio (2.06±1.21) than patients with typical ON (3.97±3.31). The Treg/Th17 ratio was significantly lower in both patient group than in control subjects (8.14±5.94; *P<*0.01 for both comparisons).

## Discussion

In present study we compared the proportion of Th17 and Treg cells and their ratio in healthy subjects and in patients with typical and atypical ON. In both typical and atypical ON patients we noted a significant up-regulation of Th17 cells, a down-regulation of Treg cells and an imbalanced Treg/Th17 ratio. These data are consistent with previous studies of Th17/Treg changes in other autoimmune disorders (7, 20–24). A balance between Th17 and Treg cells is crucial for immune homeostasis, as Th17 cells are a key player in the pathogenesis of many autoimmune diseases, and Treg cells function to restrain excessive effecter T-cell responses [17]. Many factors can influence the balance of Th17 and Treg cells, and the imbalance is often associated with disease [6, 17]. Th17 cells are induced by the presence of transforming growth factor (TGF)-beta, IL-6 and other cytokines, and play a potent pro-inflammatory role in the immune system [18]. This is accomplished through production of a range of pro-inflammatory factors including IL-17, IL-22 and granulocyte-macrophage colony stimulating factor (GM-CSF), and also by provoking neutrophil recruitment and chemokine expression [19, 20]. In contrast, Treg cells act to suppress ongoing immune responses through several direct or indirect mechanisms [21]. Treg cells are the main element for the maintenance of peripheral tolerance and growing evidence indicates that Treg cells can suppress ongoing immune reactions [6]. There are several possible mechanisms that might contribute to the loss of Treg subset in MS patients [22]. These include (i) a lower release of Treg cells from the thymus; (ii) a decrease in FoxP3 expression or; (iii) a change in Treg cell substrates [6, 23]. In animal models, Treg cells play a significant role in the development of ON by down-regulating FoxP3 gene expression, and thus its inhibitory function on Th1 and Th17 cells [24, 25]. Our findings further suggest that up-regulation of Th17 cells, down regulation of Treg cells and loss of balance of Th17 and Treg subpopulations as an important part of immune homeostasis play a crucial role in the pathogenesis of autoimmune disorders such as ON related to either MS or NMO.

We also found that the frequency of Th17 pro-inflammatory cells is significantly higher while the Treg/Th17 ratio was significantly lower in patients with atypical ON than those with typical ON. In addition to their pro-inflammatory impact within the peripheral circulation, Th17 lymphocytes may migrate through the blood-brain barrier, where they promote inflammation through CD4+ T cell recruitment and production of pro-inflammatory cytokines [26]. MS is considered as a Th1-driven autoimmune disease, and this concept has been demonstrated in animal studies of the MS model experimental autoimmune encephalomyelitis (EAE). This led to the identification of a Th17 cell subgroup, which plays an important role in MS pathogenesis [10, 11]. NMO is considered a B cell driven autoimmune disease [27, 28]. However, B cell might involve T cell in the immunological pathogenesis of NMO. In fact, B cells can activate or tolerate T cells, to help induce or suppress an immune response [29]. Researchers have recently found that one specific type of B cell can maintain Treg cells while limit the differentiation of Th17 [30]. The deficiency of B cell can affect its ability to induce Treg [31] and break the balance of Treg/Th17 [32], which may contribute to the disease process. Interestingly, two recent studies have reported that Th17 cells also increased and might play a “collaborative” role in NMO [13, 14]. In this study, our results showed that both atypical and typical ON patients have an imbalanced ratio of Treg/Th17 compared to that in control subjects, while the change in atypical ON patients is more significant. This result supports that B and T cells influence on each other and further emphasizes the role of Treg/Th17 ratio in NMOSD patients. From a clinical standpoint, the difference in Treg/Th17 ratio between atypical and typical ON suggest its value in the differential diagnosis of atypical and typical ON. Nevertheless, due to the considerable overlap between the two groups, Th cell analysis alone is less likely to be able to distinguish atypical versus typical ON patients. However, these measures could contribute to other clinical and laboratory measures to guide diagnosis and treatment, especially in the early stage of diseases.

It is possible that the increased frequency of Th17 in patients with atypical ON reflects the increased relapse frequency of atypical ON rather than the disease type itself, as repeated attacks may induce expansion in the auto-reactive Th17 cell pools. In fact, nearly half of patients in atypical ON group were recurrent, compared to that only 4 of 30 typical ON cases. To address this possibility, we re-grouped all 63 ON patients into two groups: 43 cases with first-ever attack of ON and 20 relapsing ON cases, and then compared their frequency of Th17 cells. We noted that frequency of Th17 was slightly higher in the relapsing ON group, but the difference was not statistically significant. This result suggests that the pattern of Th17 up-regulation may be a distinguishing feature between typical and atypical ON.

Immune treatment for ON is designed to target the specific immunological abnormalities present in different patient subtypes [33, 34]. Our findings of a difference in the balance of Treg and Th17 cells in patients with atypical ON compared to typical ON patients, suggest that the evaluation of pro-inflammatory and regulatory cells can add useful information in distinguishing these two conditions, and thereby guide to the most appropriate treatment strategy. Many cytokines also play important roles in the numbers modulation of Th cells, such as GM-CSF which can act as an immune modulatory cytokine to suppress autoimmunity through effects of regulatory T cells[35–37]. The treatment potential of GM-CSF for autoimmune disease has been supported in the study of animal models of autoimmune diabetes [38, 39] but and also in a patient with Myasthenia gravis[37]. GM-CSF mediated regulation of Treg cells may also provie useful in the treatment of some forms of ON, but this possibility will require further research. In future studies, we will focus on cytokines related to Th17 and Treg cells, with the hope of identifying new cellular biomarkers by which further distinguish and explore new treatment approaches for typical and atypical ON.
